# The Multiple Paths to Multiple Life

**DOI:** 10.1007/s00239-021-10016-2

**Published:** 2021-07-12

**Authors:** Christopher P. Kempes, David C. Krakauer

**Affiliations:** The Santa Fe Institute, Santa Fe, NM USA

## Abstract

We argue for multiple forms of life realized through multiple different historical pathways. From this perspective, there have been multiple origins of life on Earth—life is not a universal homology. By broadening the class of originations, we significantly expand the data set for searching for life. Through a computational analogy, the origin of life describes both the origin of hardware (physical substrate) and software (evolved function). Like all information-processing systems, adaptive systems possess a nested hierarchy of levels, a level of function optimization (e.g., fitness maximization), a level of constraints (e.g., energy requirements), and a level of materials (e.g., DNA or RNA genome and cells). The functions essential to life are realized by different substrates with different efficiencies. The functional level allows us to identify multiple origins of life by searching for key principles of optimization in different material form, including the prebiotic origin of proto-cells, the emergence of culture, economic, and legal institutions, and the reproduction of software agents.

## Introduction: Life is Everywhere

An ongoing scientific challenge has been to create a general theory of life that integrates our empirical understanding of biology with logical principles that might transcend it (Cleland 2019; Goldenfeld and Woese 2011; Goldenfeld et al. 2017; Walker et al. 2017; Walker 2017; Davies and Walker 2016; Walker et al. 2018). The search for principles that are not dependent on evolved constraints and biochemical materials has been intriguing, but has not yet led to complete theories of how to identify, quantify, or create life (Langton 1984; von Neumann 1966; Langton et al. 1992, 1994; Küppers 1990; Yockey 2005; Walker and Davies 2013). Meeting this challenge would help to address several of the most interesting questions facing the natural sciences and biology in relation to questions of generality and universality. These would include the following: (1) how do biotic mechanisms emerge from abiotic ones, (2) how can we be sure that we have found life if it is materially different from life on Earth, and by extension, how do we verify that an environment is truly lifeless, for example, in a sample of ice from Enceladus?, and (3) how do we in general understand the range of possibilities for the origin and maintenance of life?

From an evolutionary perspective, the central challenge for defining life has been the need to make a distinction between describing known evolutionary trajectories while establishing a full possibility space for life (Scharf et al. 2015). No one wants to restrict the science of life to one current realization on Earth, and prior work has exhorted origins of life researchers to study “the onset of the various organizational phenomena that we associate with the living world” (Scharf et al. 2015). We define life as the union of two crucial energetic and informatic processes producing an autonomous system that can metabolically extract and encode information from the environment of adaptive/survival value and propagate it forward through time (Krakauer et al. 2020). We provide a new perspective on the origin of life by arguing that life has emerged many times on Earth and that there are many forms of extant life coexisting on a variety of physical substrates. To help explain this position, we organize theories of life into three dominant perspectives: extant centric, history centric, and principle centric.

The *Extant-centric* approach focuses on characteristics and comparisons among existing life. This was the first focus of biology as a discipline. The *History centric* focuses on the specific evolutionary trajectories that lead to extant life including Earth’s specific origin of life and its conserved molecular traits. The *Principle centric* focuses on generalizations of life in terms of shared properties of all possible evolutionary trajectories and all possible origins of life. In each case, a focus should be interpreted as a perspective that prioritizes a certain style of work and effort.

Most agree with the need for moving from a extant- or history-centric perspective on life to a principle-centric one. However, this perspective remains under-explored—for practical reasons—and its implications have not been fully appreciated. The natural tendency is to associate life with Earth life, often restricting mechanisms supporting life to those mechanisms universal across Terran species, and, as has recently been discovered, organisms that share a common molecular ancestor. From a living-principles-first perspective, life can be defined independently from its contingent evolutionary history in terms of a suite of adaptive functions. For example, the way that macroscopic functions or features of organisms can be understood independently from their molecular or developmental mechanisms (e.g., as exemplified by the optimal properties of a variety of vascular networks of plants and mammals in Savage et al. 2004; West and Brown 2005). And by analogy, the way that effective software can be described using a logic that is different, and in many cases independent, from the details of its hardware support.

This view of life naturally opens up the possibilities for many origins in many different systems. It is also a viewpoint that revives a classic natural history perspective that categorizes biology by form and function in distinction to the modern evolutionary synthesis and molecular biology revolution that categorizes life based on lineage. While these earlier perspectives lack the unifying framework of evolution by natural selection, they recognized functional similarities and what we think of in terms of surprising biological homoplasy. We wish to generalize these similarities into ingredients for a theory of life. It could be that a focus on the evolution of life has blinded us to additional general principles of life.

For the principles-centric definition of life, there may be many origins of different types of life along an evolutionary trajectory. Some trajectories may even transition from living to non-living optimized states before giving rise to life again. We would argue that autonomous digital computers are an example of this possibility: they are created by life initially as non-living information-processing machines, but may later provide the substrate for new types of life such as through evolutionary simulations, a rather rudimentary example, and autonomous A.I., a more complicated example. Importantly, computers might eventually expand our conception of life where the human-transistor system in aggregate resides within the space of the living and where neither could persist independently akin to many extant obligate mutualisms.

Somewhat surprisingly, this approach suggests that contrary to the wide-spread belief that life has a single chemical origin and basis (history-centric), life has in fact evolved many times on Earth. Biological life at the biochemical level might have a unique provenance, but higher-level aggregations with emergent living features do not.

This forces us to distinguish between the idea of an origin and the fact of a first occurrence. This relates very naturally to the evolutionary concepts of analogy and homology. Life itself is typically considered ancestral to all of biology and thereby the ultimate homology, whereas we argue somewhat counter-intuitively that life should be thought of as analogous, or more technically as homoplastic—a set of traits that have been gained or lost independently in separate lineages over the course of evolution. Life should be thought of as a special class of convergent evolution. The multiple origins of life on Earth happen to have a common historical trajectory in LUCA. As has been noted (Walker 2020), if new life were created in a computer or in a laboratory, those specific substrates are setup by humans and create a causal link with LUCA.

Scharf et al. (2015) first presented an argument along similar lines to those here by proposing a classification of life based on historical, synthetic, and universal properties, with subfields defined by the overlap among these categories. They suggest convincingly that there could be many paths from an abiotic to biotic Earth with various potential bottlenecks, convergences, and branching points. We add the many multiple transitions from the living to the non-living and back to the living (e.g., from modern human society to solid-state devices to software-based computer viruses). And that these multiple transitions take place over a range of different levels in the life hierarchy. This implies that there is a huge richness of types of life that emerge at the principles (or universal as in Scharf et al. 2015) level, and that there are already observations of multiple origins of life on Earth when we adopt the appropriate theoretical lens, to include many products of cultural evolution. This is distinct from the perspective that characterizing life is “not in explaining the states themselves, but instead the paths” (Walker 2017) as we are interested in theories that identify the homoplasy of evolutionary endpoints.

## A Spectrum of Living Processes

The definition of life as an autonomous system that can metabolically extract and encode information from the environment of adaptive/survival value and propagate it forward through time does not make use of ideas of replication or compartmentalization but builds on recent efforts to place categorical features of life, such as individuality, onto a quantitative spectrum. The key idea is to relate life to information theoretic measures of autonomy which describes the information in a system’s past that is transmitted independently of the environment into the system’s future (Krakauer et al. 2009, 2020). In this way, life is able to encompass a variety of evolving systems, all of which can be recognized by their ability to efficiently and reliably propagate adaptive information from the past into the future. We do not define life as any evolving system because many of these will not possess autonomy or individuality but obtain their functional features entirely through external constraints and design (e.g., simple rolling stones that reduce friction through erosion or complicated examples of human built architecture from pyramids to sky scrapers).

In order to illustrate why such a theory of life needs to be foundational, consider the following taxonomic spectrum: virus, bacterium, multicellular animal, ecosystem, planet. Now ask which of these systems represents life? Almost every biologist on the planet would agree that the bacterium and the multicellular organism are living. Viruses have proven more controversail because they possess a minimum combination of autonomy in metabolic capability and coding capacity (e.g. Villarreal 2004). But all of the arguments that one uses to exclude viruses, are true of many bacterial species, such as obligate symbionts. What about individual cells in the multicellular organism, or the distinction between germline and somatic cells in those same organisms? Is it only the whole multicellular body that is alive? Can obligate predators be considered life since their metabolism is not fully autonomous? If one accepts that both cell and whole bodies are forms of life, then why wouldn’t both the individual and ecosystem be a form of life? These are all well-known debates that highlight how hard it has been to agree on the discovery of new life that possesses neither cells or bodies. The use of phosphine as a possible biosignature has already proven to be a controversial topic (e.g., Sousa-Silva et al. 2020; Cockell et al. 2020), but harder debates lurk ahead for life that could look radically different. The problem is that we cannot agree on the answers to the question of living bacterium versus virus precisely because we don’t have a fundamental theory that can quantitatively assign “livingness” to an autonomous dynamical system. The problem of relying on lists is that lists never add up to processes.

In this context it is useful to relate the idea of life to the idea of computational processes. These connections have been explored in the setting of general perspectives on life (Walker and Davies 2013). Here we are not suggesting that life is a computation but that the division of matter and logic in universal computation—what has been called “The Beginning of Infinity” (Deutsch 2011)—is precisely the type of step that needs to be taken to broaden our study of living phenomena and move beyond lists of charactersistics toward functional processes. This approach also resembles in several ways the brain-mind and genotype-phenotype binary oppositions, both of which stress the critical distinction between the material and the codical or functional domains, while allowing for significant co-dependencies between the two. We highlight several recent efforts which introduce a quantitative spectrum for various categorical features of life, such as individuality (Krakauer et al. 2009, 2020), agency (Kolchinsky and Wolpert 2018), or how much assembly an object requires (Marshall et al. 2017a, 2021; Murray et al. 2018).

### Living Across Levels

Our aim is to move toward generalized concepts and metrics for life rather than the commitment to specific characteristics or implementations (Goldenfeld and Woese 2011; Goldenfeld et al. 2017; Walker et al. 2017; Walker 2017; Davies and Walker 2016; Walker et al. 2018). Our strategy is to introduce a layered or multi-level structure for thinking about life inspired by Marr’s levels of information-processing for vision (Marr 1982) (a deeper investigation into what separates mind from brain and rather like the separation made between phenotype and genotype). Marr’s approach to distinguishing layers of information-processing (Marr 1982) is a useful analogy for illustrating the type of theory that we want to build, albeit with a greater dependence among the levels than Marr considered. Marr suggested that all information-processing architectures possess three essential levels. A computational or functional level that describes the computational problem. For example, identifying an object in a visual scene or isolating odorants in a complex biochemical mixture. A subvening algorithmic or procedural level that realizes iteratively the desired computation. For example, deep convolutional neural networks or histograms of oriented gradients. And a foundational hardware-implementation level that supports the software realizing a computation. For example, a general purpose computer, a field programmable gate array, or a graphics processing unit. All three levels are required, whereas the composition of each level can be substituted with a working alternative. Critically each of these levels interacts through fundamental constraints of architecture and thermodynamics. In Table 3, we explore how we might map between computational and biological structures and processess at each level.

For life we introduce three comparable levels: an optimisation level; a constraints level, and a material level. These are outlined in Table 1 and defined below. This approach is justified by the widely held premise that life be understood in terms of adaptive information. The hierarchy follows directly from this assumption and makes no strong claim that life is a computation. Furthermore these ontological levels should not be confused with physical-spatial levels. For example, optimization takes place at many physical levels from basic molecular mechanisms through to ecosystem engineering. In this way there can be vast numbers of nested realizations of these three levels. A few examples are listed in Table 2.

**Table 1:** Universal versus contingent theories at three levels of analysis

Level in hiearchy	Abstract theories	Biological theories
Level 3: optimization	Variational/action principles	Natural selection
		Neutral theory
		Individuality
		Agency
Level 2: constraints	Conservation laws	Allometric scaling
	Geometry and topology	Molecular packing
	Maximum entropy principle	Maximum entropy ecology and neuroscience
	Pattern formation	Reaction-diffusion morphogenesis
Level 1: materials	Chemical bonds	Protein folding
	Chemical kinetics	Gene expression dynamics

**Table 2:** How the mechanism of encapsulation can be described at three levels of analysis

Level in hiearchy	Example: “Encapsulation”	
	Computational principle	Biological mechanism
Level 3: optimization	Logical scope: local/global	Compartments/modularity
	Algorithms	Self-organization
Level 2: constraints	Type inference	Cross reactivity and specificity
	Function blocks	Regulatory circuits
Level 1: materials	Protected memory	DNA packaging
	Transistors	Enzymes

**Level 3: optimization** Life is required to maximize fitness, minimize the dissipation of metabolic free energy, efficiently encode adaptive information, and achieve strategic stability in the face of competitors (e.g., Walker and Davies 2013). The abstract frameworks at this level include the logical elements of the problem, measures of information, free energy, algorithmic complexity, and geometry. The biological theories that address these frameworks include, population and quantitative genetics, evolutionary game theory, and adaptive dynamics.

**Level 2: constraints** General principles of the physical/material world impose largely unavoidable constraints on what is being optimized at Level 3 (Schrodinger 1944; Goldenfeld et al. 2017; Goldenfeld and Woese 2011; Walker 2017; Kempes et al. 2019; Bialek 2012; Kaneko 2006; Walker et al. 2018). These include architecture (dimension, topology, conservation laws) and design principles. Biological theories that touch on these constraints include reaction-diffusion systems and pattern formation (Turing 1952), allometric scaling laws (Schmidt-Nielsen and Knut 1984; Niklas 1994; Savage et al. 2004; West and Brown 2005), canalization through regulatory interactions, mendelian segregation and its violations, the central dogma and its violations, and information aggregation mechanisms to include population coding and winner take all dynamics.

**Level 1: materials** The physical and chemical properties of matter are felt and impose limitations on the scope of Level 2 and 3. These include much of inorganic and organic chemistry, principles of kinematics, self-assembly, and biophysical laws. Biological theories at this level include the cell theory, molecular dynamics and protein folding, cell-sorting dynamics, and a variety of mesoscopic laws such as Lewis’ law (Lewis 1928).

**Fig. 1:**
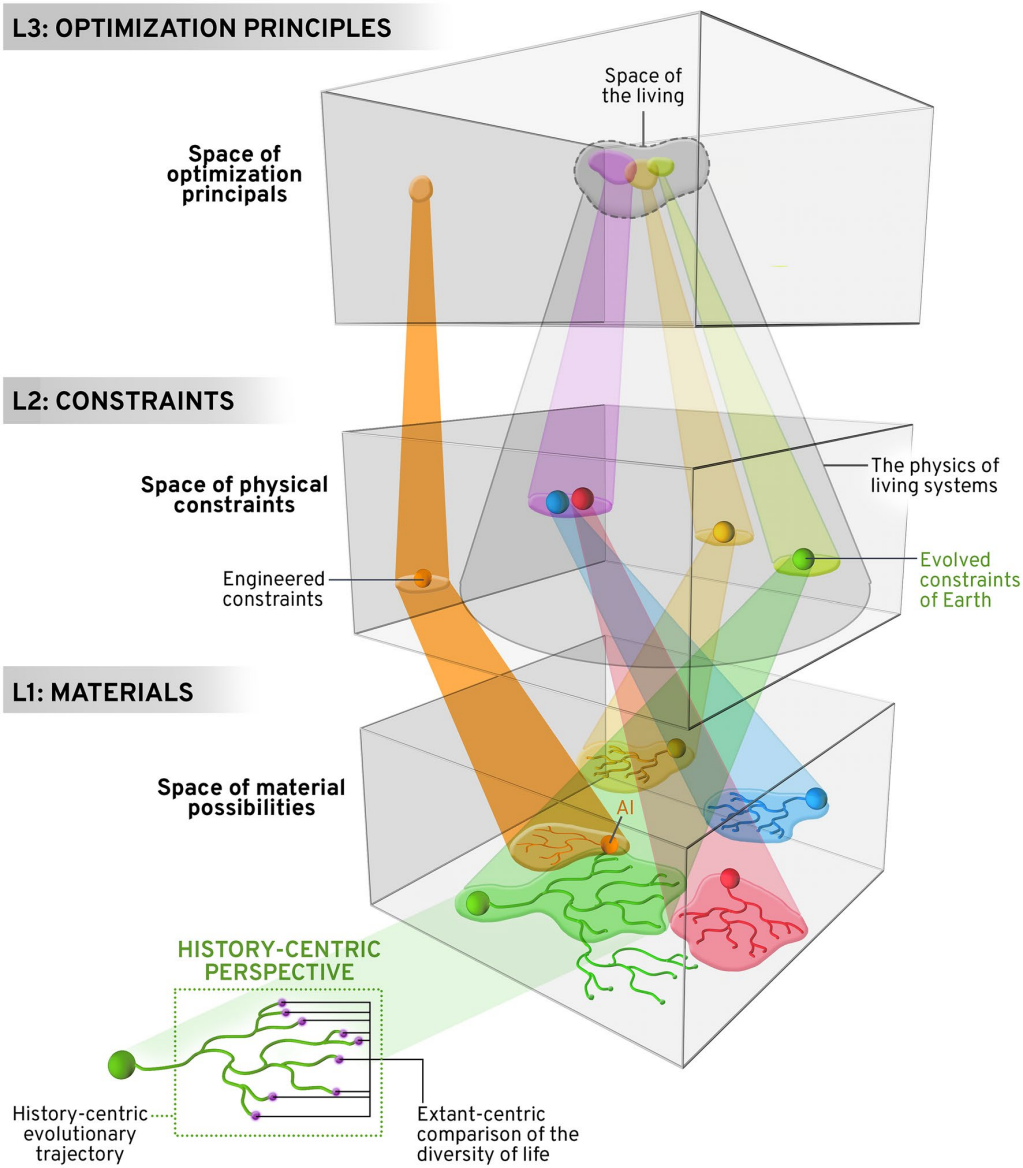
The levels of life. All life-forms follow a simultaneous trajectory within the three parallel state spaces or levels governed by material properties, constraint surfaces, and optimization principles. In L1, each phylogeny illustrates a possible evolutionary trajectory, each of which is associated with a different material origin. A history-centric approach to life equates life with a complete phylogenetic history. Extant-centric approaches seek commonality across the terminal branches of phylogenies. All points in L1 map many-to-one to points in L2. The set of points in L2 describes the space of physical constraints to include the limitations of physical laws. Evolved constraints are the sub-set of points in L2 that we describe as the physics of living systems. All points in L1 and L2 obey action or optimization principles that are defined by the set of points in L3. A small set of optimization principles such as the maximization of fitness and related concepts define the space of living action principles in L3. A principle-centric approach to life defines life in terms of the entry and restriction of a material trajectory within L1 that is constrained in L2 and only moving within the restricted space of living optimization principles in L3. Each material phylogeny in L1 is likely to be different across the universe, but can still map onto similar or identical sets of physical constraints in L2. For example, the blue and red phylogenies in L1 map onto the same set of constraints in L2. These in turn project into the space of the living in L3. In addition, living systems might produce non-living descendants. Here, we have shown in orange how a putative AI might originate from the terminal biotic branch of the green phylogeny and venture outside biology to be governed by the constraints of engineering in L2 and non-living optimization principles in L1. The reverse is also possible where abiotic materials give rise through biotechnology to new biotic life-forms. The non-unique trajectories through L1-L3 allow for the possibility of multiple life (Color figure available online; Image credit: Mesa Schumacher)

Figure 1 provides an illustration of how these three levels relate to one another, where one can see clearly interrelated evolutionary trajectories at each of the three levels. Classic evolutionary processes are realized in L1 describing the origin and diversification of lineages. All evolutionary motion in L1 is constrained by both physical conservation laws (e,g, conservation of energy) and evolved constraints (e.g., allometry) described as acceptable paths through the space of L2. And paths through L1 and L2 are guided by principles in L3 (e.g., natural selection). The extant-centric perspective on life involves inferences made from comparisons among all terminal branches of a tree, typically in L1, whereas the history-centric perspective encompasses an entire evolutionary tree in L1. L2 and L3 coarse grain the trajectories in L1 and L2 and represent a decoherent history of life—that is, families of fine-grained histories in L1 map onto fewer trajectories or points in L2 and L3.

This framework highlights the complicated connections among the levels. First, and most simply, the rates of evolution in each level will drastically differ. Typically, large changes will occur in L1 that do not change the constraints that these materials follow in L2 or the optimization principles in L3. For example, body mass might change relatively quickly across generations or taxa, but the scaling of mass with metabolism will be largely invariant. Contrariwise, small changes in L1 might lead to large shifts in L3. For example, mutations that influence the body plan or the rate of mutation can change the way that selection operates on populations. For example, a genome can undergo selection for specific GC content by selecting among synonymous codons with no change to the overall phenotype, except in the environmental requirements of the organism (e.g. Mann et al. 2010). This would be a material constraint of the environment imposing selection on the genotype where the selection for whole organism characteristics influences the genotype independently from the phenotype. The properties of the organism change but not through the genotype-to-phenotype mapping since that is preserved at the level of the amino acid coding.

L2 and L3 are most directly connected to universal abstract and mathematical principles and thus the non-living universe. L2 introduces anisotropies and biases on L3 through energetic and informational constraints, and while somewhat contingent, these will always appear in one form or another. L3 principles describes variational principles, one of which is evolution by natural selection, which is required by any form of life. And L1 is the most path-dependent, contingent, and constrained by L2 and L3.

Asserting universality at L1 would be equivalent to describing life as uniquely materially realizable, through a one-to-one mapping from L1 to L2 to L3. This would be analogous in the cultural domain to studying the evolution of one language as opposed to the evolution of languages more broadly. We need to consider some version of all three levels in order to explain the origins of Igbo, French, or Japanese, where both physical constraints of sound production and perception interact with optimization that either minimizes the time or energy to produce a signal.

A common perspective is that L1 is the most universal since it is closest to the material basis of the universe which need obey physical law. For example, Smith and Morowitz suggest that core metabolism can be understood as the most likely autocatalytic network given non-equilibrium thermodynamic considerations and environmental compositions, and that these networks are not arbitrary (Smith and Morowitz 2004; Morowitz and Smith 2007). This makes the particular combinations at this level, such as a biochemistry, exemplary of what all of life is likely to look like. However, we should be careful to extract the principles from this example—such as finding the most likely autocatalytic network conditioned on an environment—and situate those principles in the huge space of the chemical combinations of various abiotic environments and planetary conditions in order to understand the full range of material possibilities .

The most illustrative examples of this hierarchy are the connections between L1 and L2. For example, life harnesses many energetic gradients for useful anabolism via many L1 mechanisms. But all of these conform to the laws of thermodynamics and no cell will be found to contain more internal structure than can be accounted for by the total free energy available from the environment (Schrodinger 1944; Morowitz 1955). This result is well-known and illustrates a general L2 principle, in this case the laws of thermodynamics, realized on many L1 instances.

As discussed previously, some biological phenomena require explicit consideration of all three levels. For example, allometric scaling laws manifest because of specific L1 architectures under specific L2 constraints, with near perfect L3 optimization. Indeed, we expect many rich biological concepts to be defined by a “strange tangle” of the three levels, because the three levels will unavoidably coevolve. Similarly, it has been suggested that while all of life’s properties require material instantiation (L1) and obey energetic constraints (L2), the classes of informational systems that emerge (L3) in terms of optimized representation, information storage, and processing, obey more general laws independent of the underlying material aspects (Davies and Walker 2016; Walker 2017; Krakauer 2017; Krakauer and Jansen 2002). While life’s information storage and processing systems are often based on different material compositions (material level), each of these achieves greater efficiency or robustness through principles that are very general, such as error correction, sparse coding, and fractal architectures (Flack 2017; Davies and Walker 2016; Walker 2017; Krakauer 2017; Krakauer and Jansen 2002; Smith 2008; Cronin et al. 2006; Kempes et al. 2019).

Within this framework we would define life as certain hyper-regions of L3. All of which need to be able to support adaptive histories. The shape of these hyper-regions may be quite tortuous and there may be non-overlapping regions that each represent life, but the main idea is that we want to allow for scenarios where something can be defined as living with various combinations of values along the high-dimensional axes of L3. For example, something could be far out on the “intelligence” or information capacity axis, but close to the origin on the “robustness” axis and still be counted as living. Something else could have relatively minimal intelligence and have very high robustness and also be living. The goal of future work is to identify the high-dimensional surface of minimum requirements for life in L3.

### Universal Life Analogized to Universal Computation

In considering principles-centric perspectives on life, a useful analogy to make is to the idea of computation and its somewhat scale-independent features. It is perfectly accurate to say that transistors compute, CPUs compute, and computer networks compute. Every one of these performs a function, realized by an algorithm, supported by hardware. Every element in this list possesses all levels L1–L2–L3. In every case we are applying the same L3 logical principle (traditionally the Church–Turing principle (Smith 2020)) and at each level we observe a different range of L1–L2 computational power, efficiency, constraints, and range of applications (Davis 2018).

We acknowledge that without the lowest physical element many of the higher-order structures would not exist. Indeed, all of L3 can only exist on physical matter. And in particular environments defined by specific L2 constraints there may be very narrow ranges of L1 that allow an L3 to be realized. But, we do not say that only transistors compute and that all higher-order computations are merely downstream instances of the binary operations of a transistor. Every level can be understood as a computation to the extent that each level can be described in the language of L3 somewhat independently of the language of L1–L2. Not allowing for this would represent an extreme form of computational reductionism and severely limit the scope of both hardware and software engineering—your PC is every bit as much a computer as its logic gates, they just compute different functions, and the same idea generalizes to the network of computers forming an internet. This physical hierarchy is critical to effective scientific computation (Brandt 2002).

Furthermore, at this point we also distinguish, as others have (Walker and Davies 2013), between two broad classes of computer—analog and digital—which differ with respect to both hardware and software and reflect a fundamental difference of design in their use of continuous versus discrete variables and differential versus discontinuous hardware elements—differences in L1 and L2. Nevertheless, both are able to realize the property of Turing completeness (Bournez et al. 2013) the critical feature at L3.

Tracking this analogy back to life we should not confuse microscopic material properties with macroscopic logical capabilities. Or the first occurrence of a living mechanism with the origin of alternative living mechanisms. By avoiding these traps we might identify the many cases where “life” has evolved and the common conditions that support every instance. We should also be comfortable with one type of life living upon another. Proposing that cultural evolution is a type of life implemented on a collection of humans is not radically different from considering a Turing complete software or internet implemented on several Turing complete computers or even Turing incomplete computers.

A key idea is the need to focus on “the separation of physical embodiment from ability” and on whether a system can imitate cellular function (similar to another computational analogy, the Turing test) independent of size and composition (Cronin et al. 2006). While we support this perspective our argument makes a distinction between the theoretical challenge of agreeing upon and defining the set of living features and the experimental challenge of embodying specific cellular characteristics in various materials.

The computational example also helps to illustrate the interrelation of the levels. If one wants to implement a specific algorithm on a specified scale of data with a desired runtime, then there will be serious requirements for an L1 that can dissipate enough heat to avoid melting components. This could manifest as both architecture and materials solutions under a dominant L2 constraint of heat dissipation. Similarly, if cells want to avoid the error threshold at a particular temperature this may constrain which molecules can be used for information storage. There will be certain types of L1 that can only be understood from the perspective of what L3 principle they are implementing and under what L2 constraints they have been subject to. The signature of life in L1 requires conditioning on a specific L3 and L2. The trick of spotting life is to realize that a general L3 principle is being implemented on an L1 material and that the particular implementation reflects a set of L2 constraints. L1 becomes a special type of material when L3 optimizations occur under specific L2 constraints. Some of these correspondences are described in Table 2.

### Hardware, Software, Mechanisms, and Functions

Computer science is not hardware independent and is much concerned with the hardware requirements of particular algorithms, or the construction of algorithms given hardware constraints (Steiner and Athanas 2005). Distinguishing between hardware and software provides for synergies such as the use of GPUs to support deep learning architectures and training. The universality of computer languages creates a significant degree of freedom when coding a problem.

By analogy, for living systems, we might expect to see common constraints from L2 intervening on many different materials and designs. For example, network structures that most effectively distribute metabolic resources or propagate information.

This is not, however, a hard constraint or “law” of nature as different lineages have discovered different means of solving universal problems. When it comes to life the standard biological perspective tends to focus on a single or a limited number of ways of realizing particular biological functions (e.g., RNA and DNA for heredity, a universal genetic code, ATP for energy). This viewpoint draws a unique path from L1 through to L3. The standard model for biological origination is therefore rather narrow and might miss the essence of a variety of evolved biological processes by mapping function (software) too readily onto substrate (hardware). Recent advances in reprogramming the genetic code nicely illustrates the practical value of code pluralism (Chin 2017).

When we consider inheritance more broadly we find a variety of mutational and transference mechanisms that includes horizontal gene transfer, epigenetics, RNA interference, and parasexual recombination. Each represents a variety of material mechanisms for managing the tension between information preservation and adaptation (Jablonka and Lamb 2014). Thereby expanding the class of substrates that can support a given function.

Hence questions about the requirements of, for example, information storage, transmission and function are all general question about functions required by life at an appropriate level. Questions about what information-processing and storing molecules are likely to emerge out of a given geologic scenario are specific questions about the L1 hardware required to enable life.

Once we generalize this kind of dichotomy toward a hierarchy of life, we expand the number of mechanisms that might support life. For example, our designed digital computers use entirely different hardware than cells and require no evolved cellular biomolecules, yet there are considerable overlaps with life in terms of the concepts of information storage, error-prone signaling, and information-processing at L2 and L3. This overlap is one of the justifications for exploring the possibility and diversity of Artificial Life (Bedau et al. 2000).

The hardware software dichotomy is a universal feature of any systems that can be described through a functional-codical language and a physico-mechanical language. It is therefore a central concept for biology and the origin of life which, through this lens, is the manifestation of software in hardware.

### Levels, Lists, Axioms, and Generalizations

Much of the focus in the effort to define life has centered on lists of characteristics (e.g., Trifonov 2011; Kolb 2007; Benner 2010; Bains et al. 2014) , or what we refer to as, mechanical axioms. However, for most of these axioms we find exceptions, and this creates the need for more universal principles of life (Cleland 2019; Goldenfeld and Woese 2011; Goldenfeld et al. 2017; Walker et al. 2017; Walker 2017; Davies and Walker 2016; Walker et al. 2018; Kolb 2007; Cleland 2012; Benner 2010; Bains et al. 2014).

Replication is one of the most oft-cited “mechanical axioms” of life (Trifonov 2011). Additional axioms include endogenous metabolism, a container or semi-permeable interface, and the ability to evolve. If we take replication as an example of a L1 physical feature, we find that in most cases it is a proxy for the essential L2 requirement that life requires a means of forestalling entropy production (England 2015). Replication is more often than not a means of persistence (Pascal et al. 2013), including the exclusion of rivals from shared resources, or the way in which variation through imperfect copying is introduced into a population fueling natural selection. It is possible to observe all of these features without replication, and also at multiple levels of organization (Boerlijst and Hogeweg 1995). Entities perfectly able to repair regulatory circuitry and avoid death (e.g., from predation, the consumption of essential resources by competitors, or allelopathy) have no need for replication in order to persist. In a perfectly stable environment organisms don’t have the need to adapt and thus no replication requirement as a means of introducing heritable variation. It should be noted that even when adaptation is necessary it can be achieved in numerous ways—from epigenetic modification to developmental plasticity–without requiring an error-prone copying process.

A good example of repair without replication is found in the field of error-correcting codes. These make extensive use of redundancy to ensure that messages are not degraded. No computer scientist would describe redundancy based correction as replication and at no level in hardware or software does “replication” take place. Error correction is in fact a simple computation not unlike performing a summation. It is typically the Boolean “OR” function, which is the opposite of replication as these logical mappings always map from a larger redundant code, e.g., 10, 01, and 11 to the smaller output 1.

Through this example we see that entropy resistance is possible without replication and that replication is really a sub-set of persistence mechanisms associated with adapting to changing environments. Thereby we can in principle replace a key feature of two of the most common mechanical axioms of life, replication as a mechanism of stability, with a broader suite of mechanisms promoting persistence.

Similarly, and more generally, matter and energy are necessary prerequisites for life. Both material and energetic constraints imposed on organisms can be highly informative and predictive, such as through their manifestations in allometry. But neither is sufficient for determining whether something is living. After-all, material and energetic constraints are an essential part of the abiotic universe and the key ingredients for all of physical theory.

Finding the truly essential principles for a universal theory of life is a challenging and open question. For example, the process of adaptation by natural selection has been generalized to many systems including biological species, cultures, languages, and technology (Krakauer 2011). Adaptation through natural selection (L3) requires mechanisms (L1–L2) that enable information from the environment to be encoded in the memories of an agent. Memories are stored using a variety of different error-correcting codes all exploiting structured redundancies (L3) but in materials as diverse as DNA, epigenetic marks, synaptic boutons, and solid state transistors (all L1).

By combining the L3 optimisation principle of natural selection with L3 principles of error correction there emerges a new L3 principle—the error threshold (Eigen 1971). The error threshold is the maximum error rate that can be achieved in an evolving system such that the fittest lineage is preserved. This new limit can then be mapped onto any system in the class of differentially propagated objects that are mutable, provided that one understands the unique mechanisms of information storage, variability, and the utility value of the information.

In cells this list of features includes L1 properties such as biochemistry of the genome, the mutation rate during genome replication, and the total length of the genotype. In cultural evolution one can map the same dynamical process onto a set of L1 level written words, the likelihood of correctly learning and transmitting spoken words, the total size or vocabulary of the language (Nowak et al. 1999).

In this way we find a new emergent L3 principle that provides a way of grouping apparently unrelated phenomena into a class of information dynamics that obey a shared dissipation principle. This adherence to a principle could become a new axiom for a broader sense of life.

This is why we believe that the L1–L2 the mechanical axioms of life need to be expanded and generalized to principle-centric L3 descriptions in order for us to be able to understand, detect, and construct life in any context in the universe.

### From Life to Life Equivalence

Our focus is the search for a universal theory of life (Cleland 2019; Goldenfeld and Woese 2011; Goldenfeld et al. 2017; Walker et al. 2017; Walker 2017; Davies and Walker 2016; Walker et al. 2018), where we have argued that a variety of conceptual approaches are likely to broaden what we consider to be an origin of life and cause us to rethink many of the classic “mechanical axioms” of life. One of our main approaches was to compare theories of life to the theories of physics and computing. By pursuing analogies between life and computing we naturally arrive at the profound question of universality. Modern computers are both programmable (can be configured to compute a variety of functions) and universal (compute all functions in a given class). Both ideas have their origins in Turing and Church’s proofs of the Entscheidungs problem in which they show that it is not possible to solve algorithmically—i.e., compute –all statements in first order logic. In these proofs Turing and Church rigorously introduce the concepts of algorithms, computation, and their physical implementation. The idea of Turing equivalence captures the set of all computing machines that can simulate one another (bi-simulation).

The idea of bi-simulation can expand our thinking about life because to the extent that life can be described in principles that are logical and algorithmic, it is worth determining to what degree the functions of life can be supported by hardware that is universal or, by analogy with Turing equivalent, “life-equivalent”. Using the framework developed here, such an equivalence would be a principle-centric L3 description. To be concrete, multiple materials in L1 would be life-equivalent if they all mapped through L2 into the same space of the living in L3.

This is obviously a very challenging problem but there are insights both positive and negative that can be gleaned from the computational domain. Since the publication of Turing and Church’s seminal papers it has been discovered that a rather large and unlikely class of discrete dynamical systems and software systems are Turing equivalent, including The Game of Life, the computer games Minecraft and Minesweeper, most commonly used computer languages from Lisp to Python, tag systems, extended L Systems, Feynman machines, and random access machines. If such a diversity of systems are universal one might wonder what value the concept contributes to our understanding of each one.

The positive value of equivalence has been to identify the shared properties of each of these systems, to include discrete states, memory of state, programmable states, reliable state transition functions, and termination criteria. This means that at this point we have a very strong idea of how to build computers and with what level of efficiency they will operate.

The negative implication of equivalence is precisely its generality. If life is rare in the universe and our life equivalence principles indicate that many different materials can produce persistence, competition, adaptation, and evolvability, how are we to reconcile these truths?

It is our contention that the origin of life is more common and multiple than typically thought. At least at the level of equivalence principles. That is not to say that the rather unique history of life on Earth is common. The particular chemistry supporting life’s first appearance on Earth might in fact be a rather rare form of universal life machine and this is why attempts at full prebiotic synthesis have proven so challenging. We wish to make clear that the difficulty of instantiating life in the contingent biochemistry of Earth history should not be confused with the more general problem of instantiating life. In addition, it may be the case that certain systems make it much easier for life to originate than others. The human world may be a great example of this concept where intelligence, culture, social structures, and digital computers all act as ready substrates for an explosion of many new origins of new life.

## Discussion

We have argued that the emerging perspective of life is one that shifts focus from history and particular material instantiations (L1) to more general levels of shared constraints (L2) and universal classes of optimisation (L3). In line with this thinking, previous work has argued that much of our understanding of life should be focused on transitions in information, algorithms, and computational hierarchies (Walker and Davies 2013). The ultimate theory of life will certainly have ingredients from abstract theories of engineering, computation, physics (Walker 2017), and evolution, but we expect will also require new perspectives and tools, just as theories of computation have.

Once materials and constraints at L1–L2 come into existence capable of supporting L3, then L3 can recruit new kinds of L1–L2 to generate diverse forms of life. For example, artificial life is supported by radically different materials and constraints than organically evolved life. However, organically evolved life came first, i.e, the first L3 needed to be supported by organic macromolecules. This suggests a possible theory of accelerating life production, whereby new L3 levels arrive at an increasing pace. There is of course evidence for this. Material culture is relatively recent in biological terms: stone tools first appeared just under two million years ago, cave art around seventy thousand years ago, pre-cuneiform writing around five thousand years ago, and movable type around five hundred years ago. Boolean logic was invented less than two hundred years ago and the first universal computer was built just over seventy years ago. The birth of computers obviously required all of these prior cultural inventions to exist to be at all possible. The history of culture is a history of dependency, so called implicational scaling, and one of acceleration.

Our claim is that we will be able to tell that we have a new theory of life when it is able to reveal to us many origins and many types of life. It should be able to highlight life as the ultimate homoplasy (convergence) rather than homology, where life is discovered repeatedly from many different trajectories. It should be able to define what is shared among all of the living endpoints of many trajectories and be able to assign to any system or process a degree of “livingness”. At this point we do not know whether our framework implies that the space of the living in L3 has rather blurry boundaries, or whether the boundary is sharp, and degrees-of-livingness should be measured in terms of their distance to this boundary. We suspect that these boundaries will depend very much on the nature of the changes in L1. For example, a fatal knockout mutation in L1 causes a discontinuous change in L3. Either way, many recent efforts have begun to construct metrics for a spectrum of living characteristics. For example, quantifications of the assembly required for objects (Marshall et al. 2017a, 2021; Murray et al. 2018), information theoretic decompositions of individuality (Krakauer et al. 2020), causal boundaries of living systems (Marshall et al. 2017b), physical assessments of the agency of systems (Kolchinsky and Wolpert 2018), and the processes of acquiring functional information (Lachmann and Walker 2019) have all been recently proposed and have promising future directions. Similarly, other recent efforts have elucidate general constraints at L2, such as the connection between fundamental energetics and cellular physiology and evolutionary processes (Savage et al. 2004; West and Brown 2005; DeLong et al. 2010; Lane and Martin 2010; Kempes et al. 2012; Lynch and Marinov 2015; Kempes et al. 2016, 2019; Ilker and Hinczewski 2019).

It is from the astrobiological perspective that our arguments in favor of principles will demonstrate their greatest value as we search for evolutionary sequelae off-world. These are likely to include, principles as wide-ranging as self-organized criticality, characteristics of highly optimized network structures, evidence for the maximization of mutual information, the emergence of multiple characteristic adaptive times scales, and wide-spread structural convergences.

## Table Descriptions

In the following tables we consider the interpretation of each of the three levels of analysis for living systems through (1) General theories and abstractions versus Biological Theories; (2) the relationships between computational principles and biological mechanisms; and (3) the rank order of emphasis placed on each level by different fields and disciplines, from highest emphasis = 1 to lowest emphasis = 3. In the final column of Table 3, physical theory ranks $x=3;y=2$ , whereas biophysical theory ranks $x=2;y=3$.

**Table 3:** Disciplinary attitudes to the three levels of analysis

Level in hiearchy	Typical rank emphasis of research area (1 is highest importance)				
	Natural history	Molecular biology	Mathematical biology	Biochemical origins	Principles of life
Level 3: optimization	2	3	1	2	1
Level 2: constraints	1	2	2	3	x
Level 1: materials	3	1	3	1	y
